# Baseline HIV-1 tropism prediction in advanced immune suppressed patients: evidence of CXCR4 viruses in IDUs infected with recombinant forms

**DOI:** 10.1186/1471-2334-14-S4-O20

**Published:** 2014-05-29

**Authors:** Simona Paraschiv, Ionelia Bâțan, Leontina Bănică, Iulia Niculescu, Dan Oțelea

**Affiliations:** 1 National Institute for Infectious Diseases “Prof. Dr. Matei Balş”, Bucharest, Romania

## 

Until 2011, the main risk factor for the spread of HIV-1 in Romanian adult population was heterosexual contact. More recently, the number of HIV-1 new cases among IDUs significantly increased. This new subepidemic is characterized by circulation of particular forms of infective strains (CRF14_BG), high prevalence of HCV co-infections and infective endocarditis. Genotypic methods are currently used for testing viral tropism in HIV infected patients. Deep sequencing proved to have much higher sensitivity than population sequencing in detecting minority - CXCR4 tropic viruses. Previous studies suggested that the presence of CXCR4 phenotype at baseline is frequently associated with a faster disease progression. The aim of the study was to evaluate the viral tropism at the moment of HIV diagnostic in IDU patients.

We have analyzed sequences from 19 IDUs that presented low CD4 counts (<200 cells/cmm) and/or CDC stage C when HIV-1 infection was diagnosed. They were compared with strains from 24 heterosexuals diagnosed at the same time with the IDUs were included in the study. RT PCR was performed to amplify the V3 loop. Population sequencing was done using BigDye chemistry and 3500 Genetic Analyzer. Deep-sequencing was performed on the GS Junior 454 sequencing platform and AVA software was used to analyze the output sequences. The tropism prediction was assessed by geno2pheno_[coreceptor]_ bioinformatic algorithm and subtyping with REGA tool version 2.0.

The IDU group was mainly infected with recombinant forms: CRF14_BG and recombinants between F1 and CRF14_BG (68.4%, 13/19); the heterosexuals had F1 subtype viruses (95.8%, 23/24). CXCR4 tropism was associated with IDUs and in particular with CRF14_BG (p=0.0027). All the CRF14_BG were X4 by population sequencing. Furthermore, when tested with deep sequencing the viral populations of CRF14_BG samples were exclusively X4 (no minority R5 populations). Dual tropic (CCR5 and CXCR4) populations were more frequent in F1 samples isolated from heterosexuals and predicted as X4 by population sequencing. We found concordance between the predictions of the two methods. In the heterosexual group both techniques predicted mainly CCR5 viruses.

CXCR4 tropic CRF14_BG viruses were very common in IDUs at baseline. This may contribute to faster disease progression in this population than in heterosexuals infected with the F1 CCR5 tropic strains.

